# Sulforaphane Modulates the Inflammation and Delays Neurodegeneration on a Retinitis Pigmentosa Mice Model

**DOI:** 10.3389/fphar.2022.811257

**Published:** 2022-03-01

**Authors:** Antolín Canto, Javier Martínez-González, María Miranda, Teresa Olivar, Inma Almansa, Vicente Hernández-Rabaza

**Affiliations:** Department of Biomedical Sciences, Faculty of Health Sciences, Institute of Biomedical Sciences, Cardenal Herrera-CEU University, CEU Universities, Valencia, Spain

**Keywords:** neuroinflammation, glial cells, rd10, sulforaphane, retinitis pigmentosa

## Abstract

The term retinitis pigmentosa (RP) describes a large group of hereditary retinopathies. From a cellular view, retinal degeneration is prompted by an initial death of rods, followed later by cone degeneration. This cellular progressive degeneration is translated clinically in tunnel vision, which evolves to complete blindness. The mechanism underlying the photoreceptor degeneration is unknown, but several mechanisms have been pointed out as main co-stars, inflammation being one of the most relevant. Retinal inflammation is characterized by proliferation, migration, and morphological changes in glial cells, in both microglia and Müller cells, as well as the increase in the expression of inflammatory mediators. Retinal inflammation has been reported in several animal models and clinical cases of RP, but the specific role that inflammation plays in the pathology evolution remains uncertain. Sulforaphane (SFN) is an antioxidant natural compound that has shown anti-inflammatory properties, including the modulation of glial cells activation. The present work explores the effects of SFN on retinal degeneration and inflammation, analyzing the modulation of glial cells in the RP rd10 mice model. A daily dose of 20 mg/kg of sulforaphane was administered intraperitoneally to control (C57BL/6J wild type) and rd10 (Pde6brd10) mice, from postnatal day 14 to day 20. On postnatal day 21, euthanasia was performed. Histological retina samples were used to assess cellular degeneration, Müller cells, and microglia activation. SFN administration delayed the loss of photoreceptors. It also ameliorated the characteristic reactive gliosis, assessed by retinal GFAP expression. Moreover, sulforaphane treatment regulated the microglia activation state, inducing changes in the microglia morphology, migration, and expression through the retina. In addition, SFN modulated the expression of the interleukins 1β, 4, Ym1, and arginase inflammatory mediators. Surprisingly, M2 polarization marker expression was increased at P21 and was reduced by SFN treatment. To summarize, SFN administration reduced retinal neurodegeneration and modified the inflammatory profile of RP, which may contribute to the SFN neuroprotective effect.

## Introduction

Retinitis pigmentosa (RP) englobes a range of genetic retinal diseases, which cause progressive degeneration of the photoreceptor retinal layer. It has been described that the RP induces first a cellular degeneration of rods, followed by cone degeneration (Hartong et al., 2006). This histopathology pattern produces night blindness, followed by tunnel vision, and finally a total vision loss (Hamel, 2006; Hartong et al., 2006). The RP is the main cause of blindness in young people and the main cause of hereditary blindness all over the world (Hartong et al., 2006). There is no effective cure despite this disease affecting almost 2 million people all over the world (Farrar et al., 2002; Hartong et al., 2006).

Retinal neurodegeneration induces an inflammation reaction, which has been proposed as a crucial mediator of the RP degeneration process. Neuroinflammation is characterized by vascular and glial reactions, which are translated into the production of inflammatory mediators and physiologic and morphologic glial cell activation, including the Müller cells and microglia. Furthermore, chronic inflammation plays a deleterious effect on the retinal function, as has been shown in several studies (Zabel et al., 2016). A genetic disease, such as RP, forces a genetic approach, a possibility that is not currently accessible. The reduction of inflammation research emerges as an interesting field and may provide insights into the mechanisms underlying RP development. It may also help to find new therapeutic targets to reduce the evolution and deleterious effects of RP (Fahim, 2018).

Sulforaphane (SFN) is an antioxidant natural compound (1-isothiocyanate-4-methylsulfonylmethane), found in cruciferous vegetables, that has shown antioxidant properties (Li et al., 2019; Vanduchova et al., 2019). SFN antioxidant properties are mediated by the regulation of the Nrf2 (nuclear factor erythroid 2-related factor 2) pathway. Nrf2 is a transcription factor that modulates the transcription of several antioxidant genes through its interaction with the antioxidant response elements (ARE) complex. SFN induces the Nrf2 action, increasing its cellular expression, as well as the activation and nuclear translocation of Nrf2. Nrf2 cellular level is low in health and unstressed cells, mainly by the action of Kelch-like-ECH-associated protein 1 (KEAP1), which regulates Nrf2 by ubiquitylation and proteasomal degradation. Further to the initial role of SFN as an antioxidant inductor, recent data indicated that the Nrf2/ARE pathway is involved in the regulation of inflammation, including neuroinflammation, and several metabolic derangements (He et al., 2020). These new beneficial actions of Nrf2, have replaced the focus over SFN. Recently, SFN has gained interest as a potential neuroprotective natural agent, including its possible role as an anti-inflammatory target in several neurodegenerative diseases (Li et al., 2019).

Macrophage activation is a hallmark of chronic inflammation. However, the activation of macrophages displays a gradient between proinflammatory and anti-inflammatory states, based on different inflammatory mediator profiles, as well as different proliferation, migration, and cellular morphological patterns. This gradient is dynamic, and the regulation of the macrophage activation states drives the inflammation progression and evolution (Crain et al., 2013).

Microglia cells, resident neural macrophages, show two main poles of activation, the M1 (classical, proinflammatory) and M2 (alternative, anti-inflammatory) (Crain et al., 2013). The anti-inflammatory properties of SFN have been linked with the microglia activation state regulation (Townsend and Johnson, 2016). It has been reported that SFN modulates the microglia activation states in different animal models studies. Specifically, SFN may induce the swap toward the alternative M2 anti-inflammatory state, suggesting that the beneficial SFN effects could be explained, in part, by the regulation of the microglial activation (Townsend and Johnson, 2016).

During the last two decades, SFN effects over the retina have been posted in a shortlist of spotlight studies, through *in vitro* and *in vivo* research, highlighting the SFN neuroprotective features, including beneficial reports in works on epithelial cells and oxidative stress (Dulull et al., 2018), microglial activation (Subedi et al., 2019), photoreceptor degeneration (Pan et al., 2014), retinal pigment epithelial cell degeneration, including human retinal pigment epithelium cells (ARPE-19) (Gao and Talalay, 2004; Ye et al., 2013), and models of retinal ischemia–reperfusion (Pan et al., 2014; Gong et al., 2019), as examples. Mostly all the publications pointed out the neuroprotective SFN potential through the regulation of antioxidant pathways. Recently, new publications have explored the inflammation role of SFN in diabetic retinopathy, highlighting the inhibition of the inflammasome as a mechanism (Li et al., 2019). Regarding RP, SFN has been tested in the rd10 animal model, showing a reduction in cellular degeneration and recovery of cellular retinal response, tested by electroretinography, the inhibition of reticular stress being one of the mechanisms suggested (Kang and Yu, 2017).

All these initial studies tend to confirm the SFN potential as a neuroprotective agent, but still, there are many issues to elucidate, such as the possible SFN role over glia regulation and chronic inflammation progression. In this study, we have explored the SFN effect in an animal model of RP, the rd10 mice (Pde6brd10). We have analyzed the effects of a continuous daily SFN treatment on cellular degeneration and neuroinflammation and focused our cellular analysis on the microglia activation profile. Understanding the modulation of inflammation through the progression of RP, with a special focus on microglia activation, will help to understand the disease and evaluate potential treatments.

## Material and Methods

### Experimental Design

**FIGURE 1:**
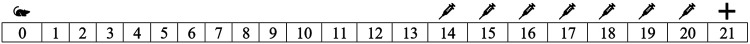
Experimental chronogram. Intraperitoneal injections, with sulforaphane (SFN) or saline, were done to the experimental groups, Control, and RD10 during the period illustrated in the figure.

C57BL/6J wild-type and Pde6brd10 mice were used, as control and RP animal models, respectively. Mice were housed in the facilities of the Research Unit of the Department of Biomedical Sciences of the CEU—Cardenal Herrera University. The animals were kept in cages under controlled conditions of temperature (20°C) and humidity (60%) and constant light–dark cycles of 12 h. The animals had free access to water and a standard diet manufactured and distributed by Harlan Ibérica S.L. (Barcelona, Spain). The body weight of the experimental animals was monitored and recorded throughout all the experiments. No differences between the body weight of the experimental groups were observed (data not shown). Handling and care of the animals were approved by the ethical committee of the CEU—Cardenal Herrera Universities (General Department of Agriculture, Livestock and Fisheries, Government of Valencia, Spain, code:2019/VSC/PEA/0040) and followed the “Declaration for the use of animals in ophthalmological and vision research” (ARVO; Association for Research in Vision and Ophthalmology). Day of birth was considered as postnatal day 0 (P0). P14 was chosen as the day to start intraperitoneal SFN treatment (20 mg/kg weight; sulforaphane was dissolved in sterile saline solution and administered using insulin-size syringe). The dose was selected as a result of preliminary experiments performed by our group and revision of the current field (Greaney et al., 2016; Kang and Yu, 2017; Hernández-Rabaza et al., 2019). To evaluate the SFN effect, each mouse received intraperitoneal SFN administration on consecutive days. The last day of treatment was P20. Mice were euthanized on P21, 24 h after the last sulforaphane dose administration. The administration program is presented in Figure 1. Note that SFN is quickly metabolized, and its body level decreases significantly around 24 h (Clarke et al., 2011).

All the experimental solutions were administered by intraperitoneal injections. Four experimental mice groups were used, Control Saline (C57BL/6J wild-type mice treated with saline), Control SFN (C57BL/6J wild-type mice treated with sulforaphane), RD10 Saline (Pde6brd10/J mice treated with saline), and RD10 SFN (Pde6brd10/J mice treated with sulforaphane). The number of mice used in each experimental group was at least *n* = 5. Our studies were completed on both male and female populations.

### Histological and Immunofluorescence Studies

Eyeballs were fixed by immersion in 4% paraformaldehyde for 2 h, then three washes were performed with 0.1 M phosphate-buffered saline pH 7.2 (PBS) for 10 min. Later, they were cryoprotected using PBS–sucrose in increasing sucrose concentrations (10%–20%–30%) at 4°C.

Afterward, 8-μm-thick retinal sections were obtained by a Leica CM 1850UV Ag protect cryostat, (Leica Microsystems SLU, Barcelona, Spain) on adhesion slides (SuperFrost, Thermo Fisher Scientific, Braunschweig, Germany) and kept at −20°C until their use.

### Histological Study of Retinal Degeneration

Retinal degeneration was studied histologically using hematoxylin and eosin (H&E) staining on the four experimental groups.

The quantification method consisted of the measurement of the outer nuclear layer (ONL) thickness in terms of the number of cell rows. To this end, a Leica DM 2000 microscope with ×40 magnification was used, with the software Leica Application Suite version 2.7.0 R1 (Leica Microsystems SLU, Barcelona, Spain) for obtaining the photos of each retina, followed by a manual cell counting by two experienced observers in double-blind conditions.

This procedure was performed in three different retinal histological sections for each eye and three different regions in each section. These regions were central retina (near the optical nerve), mid peripheral retina (between the central retina and far peripheral retina), and far peripheral retina (near the ora serrata). The distance between each retinal region was 500 µm.

### Terminal Deoxynucleotidyl Transferase Assay

The TUNEL assay was performed with an *in situ* cell death detection kit (Roche Diagnostics, Mannheim, Germany) as described in Benlloch-Navarro et al. (2019). To analyze retinal TUNEL-positive cells, images were taken with a Nikon DS-Fi1 camera attached to a Leica DM 2000 microscope, with the software Leica Application Suite version 2.7.0 R1 (Leica Microsystems SLU, Barcelona, Spain).

The TUNEL-positive cells were counted manually, by a blind experimenter, in the ONL of three different regions of the retina: central retina, mid peripheral, and far peripheral retina. The distance between each retinal region was 500 µm. TUNEL-positive cells from three retinal sections were counted for each animal of each group, one area per region. The count was taken at ×20 magnification, and the number of cells was referred to the area of the ONL, which was used. This was done with the help of the software ImageJ Fiji 1.5.3.

### Retinal Immunofluorescence Studies

Immunofluorescent staining procedures were performed on retinal cryosections that were rehydrated in PBS and merged for 1 h at room temperature (RT) with blocking solution: 10% of normal goat serum in PBS–BSA 1% and Triton 0.1%. Afterward, they were incubated overnight at 4°C with primary antibodies: anti-glial fibrillary acidic protein (anti-GFAP) (1:200, Dako Cytomation, Denmark), anti-ionized calcium-binding adaptor molecule 1 (Iba1; 5 μg/ml, Abcam, Cambridge, United Kingdom), anti-interleukin 4 antibody (IL-4; 5 μg/ml, Abcam, Cambridge, United Kingdom), anti-interleukin-1β (IL1β; 1:100, Abcam, Cambridge, United Kingdom), anti-liver arginase antibody (arginase; 5 μg/ml, Abcam, Cambridge, United Kingdom), and anti-chitinase three-like protein three antibodies (CHI3L3, also named Ym1; 20 μg/ml, Abcam, Cambridge, United Kingdom). The next day, sections were washed and incubated for 1 h in darkness at RT with the fluorescence-conjugated secondary antibody Alexa Fluor 488 (Invitrogen, Life Technologies, Madrid, Spain). Sections were mounted with Vectashield with DAPI (Vector, Burlingame, CA, United States).

In the case of double immunofluorescence staining, after the incubation with blocking solution, tissue sections were incubated overnight with Iba1 antibody. Next, incubation with a secondary antibody (Alexa Fluor 488 or Alexa Fluor 568) for 1 h was performed. Afterward, incubation overnight with IL1β or IL4 antibodies followed by 1 h of secondary antibody (Alexa Fluor 488 or Alexa Fluor 568) incubation was performed. Ultimately, sections were also mounted with Vectashield with DAPI.

Retinal images were taken with a Nikon DS-Fi1 camera attached to a Leica DM 2000 microscope, with the Leica Application Suite version 2.7.0 R1 program (Leica Microsystem SLU, Barcelona, Spain). Representative images were taken of the central retina, mid peripheral, and far peripheral retina regions (×20 magnification). The distance between each retinal region was 500 µm. Finally, images were quantified with the help of the software ImageJ Fiji 1.5.3. It must be noticed that area is quantified as arbitrary units (AU) (656 AU represents 100 µm).

To evaluate changes in reactive gliosis, the percentage of area occupied by the GFAP antibody labeling was measured throughout the retina. Regarding Iba1 expression, quantification was done in three different ways. First, the number of cells in each layer and the whole retina were measured. Second, it was performed as a migration index. Last, a morphological analysis of the Iba1 cells was performed.

In order to evaluate microglial activation, we counted the total Iba1-positive cells in the whole retina and each layer. This value was divided by the area of each layer. Results were expressed in percentage regarding the maximum value of Iba1 expression in each layer. Regarding the evaluation of microglial migration to the damaged areas, we measured the migration index (MI), which is defined following the method described by Martínez-Fernández de la Cámara et al. (2015), as the number of Iba1-positive cells weighted according to the retinal layer where they are located [MI = ∑ (number of Iba1-positive cells in each layer × layer weighted factor)/total number of Iba1-positive cells in the section]. The layer weighted factor was 1, 25 for the outer nuclear layer (ONL), 1 for the outer plexiform layer (OPL), 0, 75 for the inner nuclear layer (INL), 0, 5 for the inner plexiform layer (IPL), and 0, 25 for the ganglion cell layer (GCL). Finally, regarding the Iba1 morphology study, to quantify the number and length of cellular branches, fluorescence photomicrographs were converted into skeletonized images and analyzed using the ImageJ Fiji 1.5.3. Analyze Skeleton and FracLac were used according to the method described by Young and Morrison, (2018) for this purpose. In this case, data are expressed in arbitrary units (AU) per nucleus.

To evaluate IL1β expression, including both the mature and pro-form of IL1β, the percentage of area occupied by the IL1β antibody labeling was measured throughout the retina. To evaluate IL4 expression, Ym1 expression, and arginase expression, positive cells were counted manually in the retina, by an experimenter in blind conditions. Three retinal sections were counted for each animal of each group, and the number of positive cells was referred to the area of the retina which was used. For the colocalization quantification, the amount of IL1β-Iba1-positive cells was manually counted, and the results were expressed according to the amount of Iba1-positive cells.

### Statistical Analysis

The results are presented as mean values ± standard deviation. Each percentage change was performed using the RD10 SFN group reduction/increase in comparison with RD10 SAL results. To ensure the normal distribution of the groups, the Shapiro–Wilk test was performed. Variance homogeneity was determined by Levene’s test of variance homogeneity. The two-way analysis of variance (ANOVA) was used. When the ANOVA indicated a significant difference, the Bonferroni test was performed. SPSS software package version 27.0 was used. In every case, it was assumed that a p-value lower than 0.05 is significant.

## Results

### Sulforaphane Administration Reduces Retina Degeneration

Cellular retinal degeneration was assessed with a double cellular analysis. First, histological analysis of the thickness of the outer photoreceptor layer, stained with H&E, and second a quantification of positive TUNEL cells were performed. The cellular counting was realized in three different retinal regions with the optic nerve position as reference, called far and mid periphery and central nerve regions.

**FIGURE 2:**
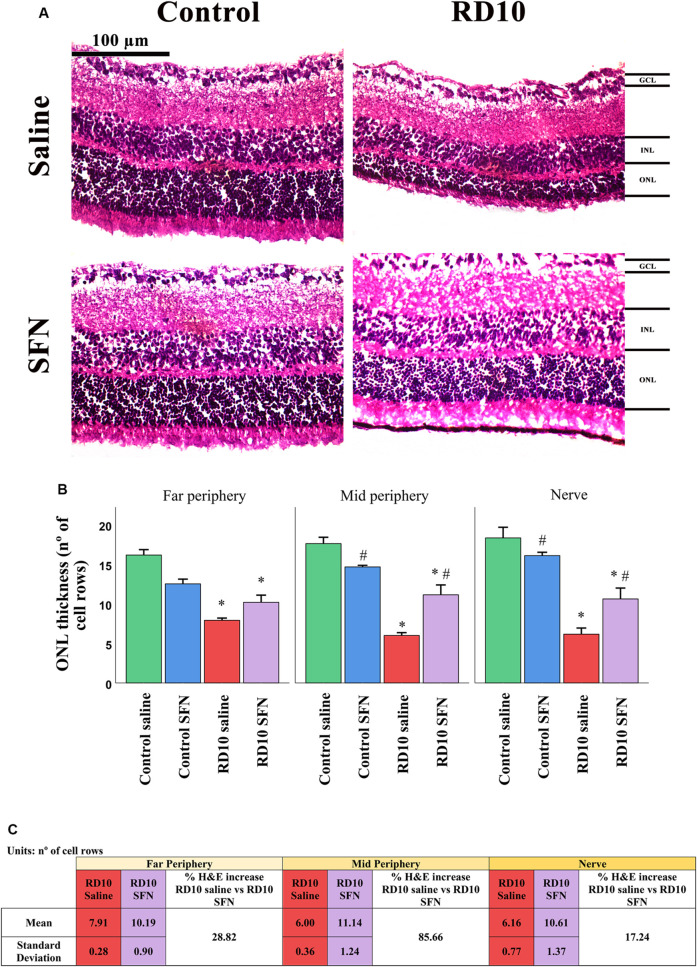
Hematoxylin and eosin (H&E) cell analysis. **(A)** Nerve region H&E images of the different animal groups in the experiment. **(B)** The number of cell rows in the outer nuclear layer (ONL) in the different studied regions. *Differences between Control Saline vs. RD10 Saline or Control SFN vs. RD10 SFN (p-value <0.05). #Differences between Control Saline vs. Control SFN or RD10 Saline vs. RD10 SFN (*p*-value <0.05). **(C)** Effect of SFN on RD10 mice H&E cell count. RD10 experimental groups: mean and standard deviation and H&E cell percentage expression differences.

H&E results indicate that all the RD10 groups, treated with or without SFN, show a significant reduction in the number of row cells in the ONL regarding the Control groups (in all the retina areas analyzed, Control Saline vs. RD10 Saline *p*-value <0.000, Control SFN vs. RD10 SFN, *p* < 0.001). However, the RD10 SFN group suffered a significant minor cellular degeneration, particularly pronounced in the mid and nerve periphery regions, where statistically significant differences were found between the RD10 Saline and RD10 SFN groups (*p* < 0.000), suggesting a delay in the neurodegeneration. The H&E results are illustrated in Figure 2, including the mean increase in RD10 SFN in comparison with the RD10 Saline group (Figure 2C).

**FIGURE 3:**
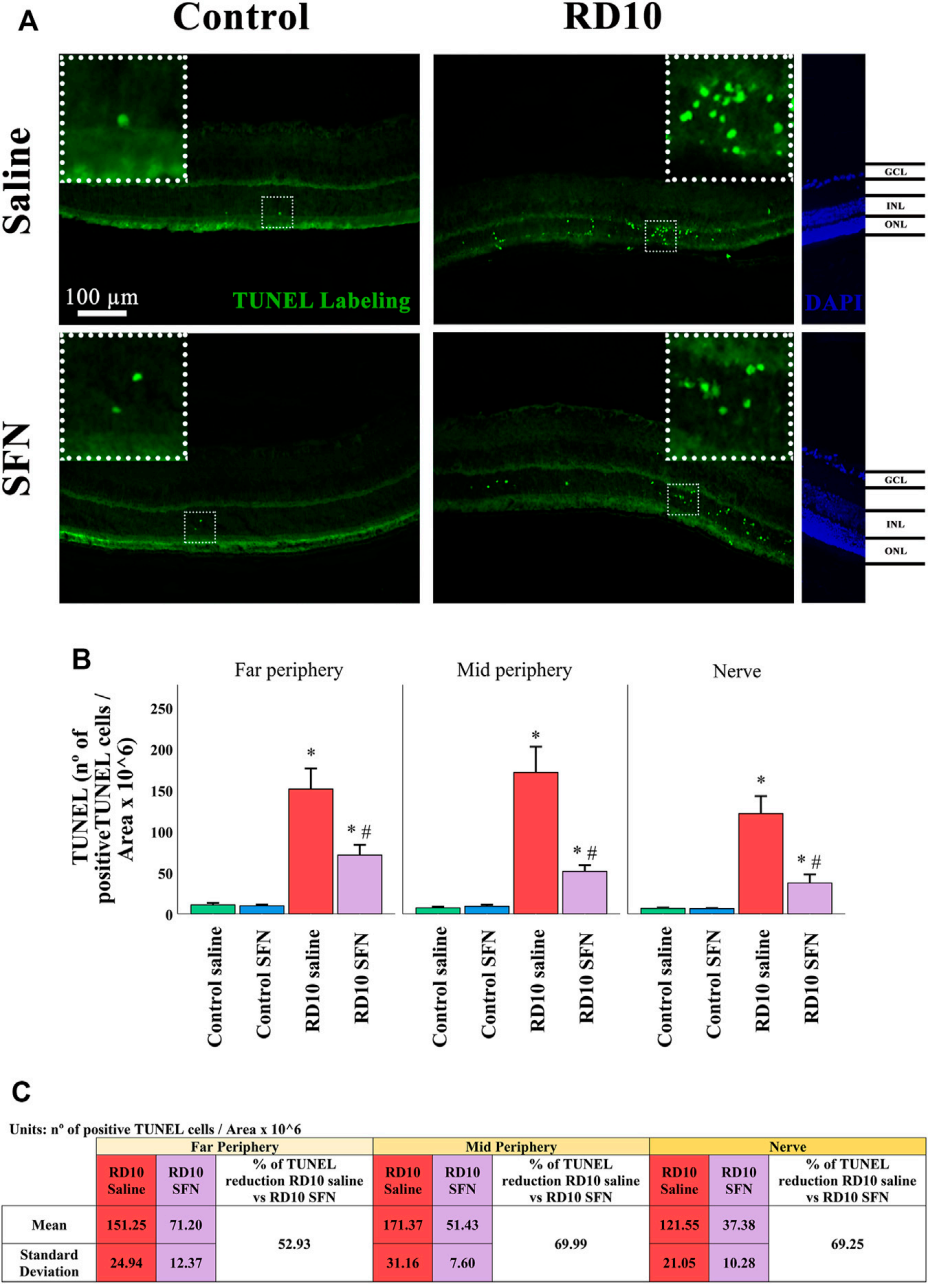
Terminal deoxynucleotidyl transferase (TUNEL) cellular stain assessment. **(A)** Nerve region images of TUNEL immunofluorescence of the different groups in the experiment; the positive cells are pointed by white arrows. **(B)** The number of TUNEL-positive cells divided per area and multiplied per 10^6^. *Differences between Control Saline vs. RD10 Saline or Control SFN vs. RD10 SFN (*p*-value <0.05). #Differences between RD10 Saline vs. RD10 SFN (*p*-value <0.05). **(C)** Effect of SFN on RD10 mice TUNNEL cell count expressed by the mean, standard deviation, and percentage reduction of RD10 SFN with respect to the RD10 Saline.

In addition to the H&E data, immunofluorescence analysis of the TUNEL cellular death marker confirms the neuroprotective action of SFN. The results indicate that all the RD10 groups, treated with or without SFN, show a significant increase in TUNEL-positive cells regarding the Control groups (in all the retina areas analyzed, Control Saline vs. RD10 Saline, p-value <0.000, Control-SFN vs. RD10 SFN, *p* < 0.000). However, the RD10 SFN group showed a significant reduction concerning the RD10 Saline group, in the far periphery (*p* < 0.000), in the mid periphery (*p* < 0.000), and in the nerve (*p* < 0.000) regions. The TUNEL results are illustrated in Figure 3, including the mean decrease in RD10 SFN in comparison with the RD10 Saline group (Figure 3C).

### Sulforaphane Reduces the Glial Cell Activation

Immunohistochemistry data indicated an inflammation process on the RD10 Saline group, which was reverted by the SFN treatment. This affirmation is based on the cellular analysis of the glial cell activation, including both Müller cells and microglial cells.

**FIGURE 4:**
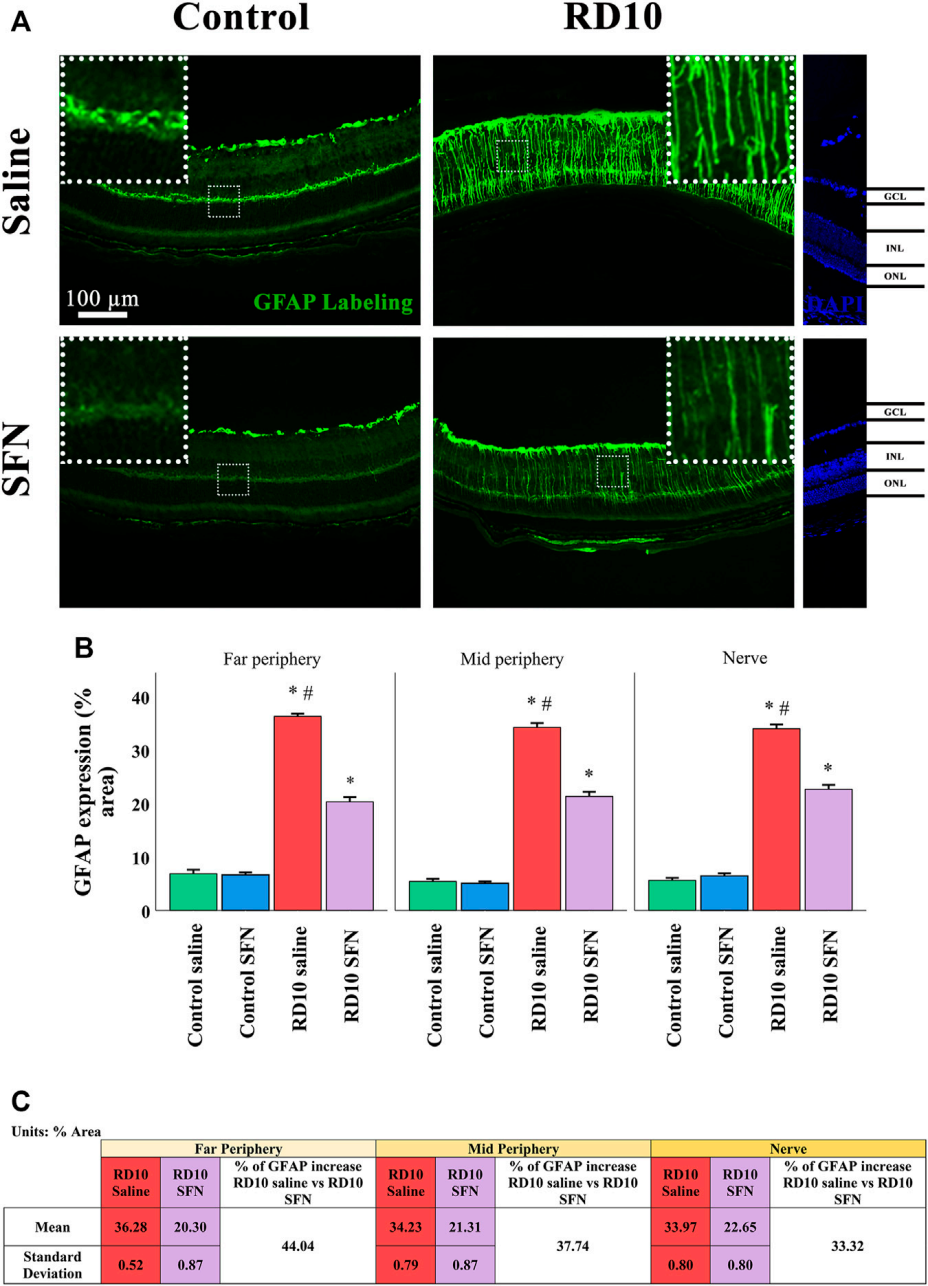
GFAP cellular analysis. **(A)** Nerve images of GFAP immunofluorescence of the different groups in the experiment. **(B)** GFAP percentage. *Differences between Control Saline vs. RD10 Saline or Control SFN vs. RD10 SFN (*p*-value <0.05). #Differences between RD10 Saline vs. RD10 SFN (*p*-value <0.05). **(C)** Effect of SFN on RD10 mice GFAP mark expressed by the mean, standard deviation of the RD10 groups, and percentage reduction of RD10 SFN vs. RD10 Saline.

Müller cell expression was analyzed with the GFAP marker, which was increased in the RD10 Saline in comparison with both control groups (in all the regions analyzed, Control Saline vs. RD10 Saline, *p*-value <0.000, Control SFN vs. RD10 SFN, *p* < 0.000). This effect was reduced by the SFN treatment in the RD10 group in the three regions analyzed (significant differences with respect to the comparison of RD10 Saline and RD10 SFN groups, *p* < 0.000, far periphery; *p* < 0.000, mid periphery; and *p* < 0.000, nerve region). The GFAP results are illustrated in Figure 4, including the mean decrease in RD10 SFN in comparison with the RD10 Saline group (Figure 4C).

**FIGURE 5:**
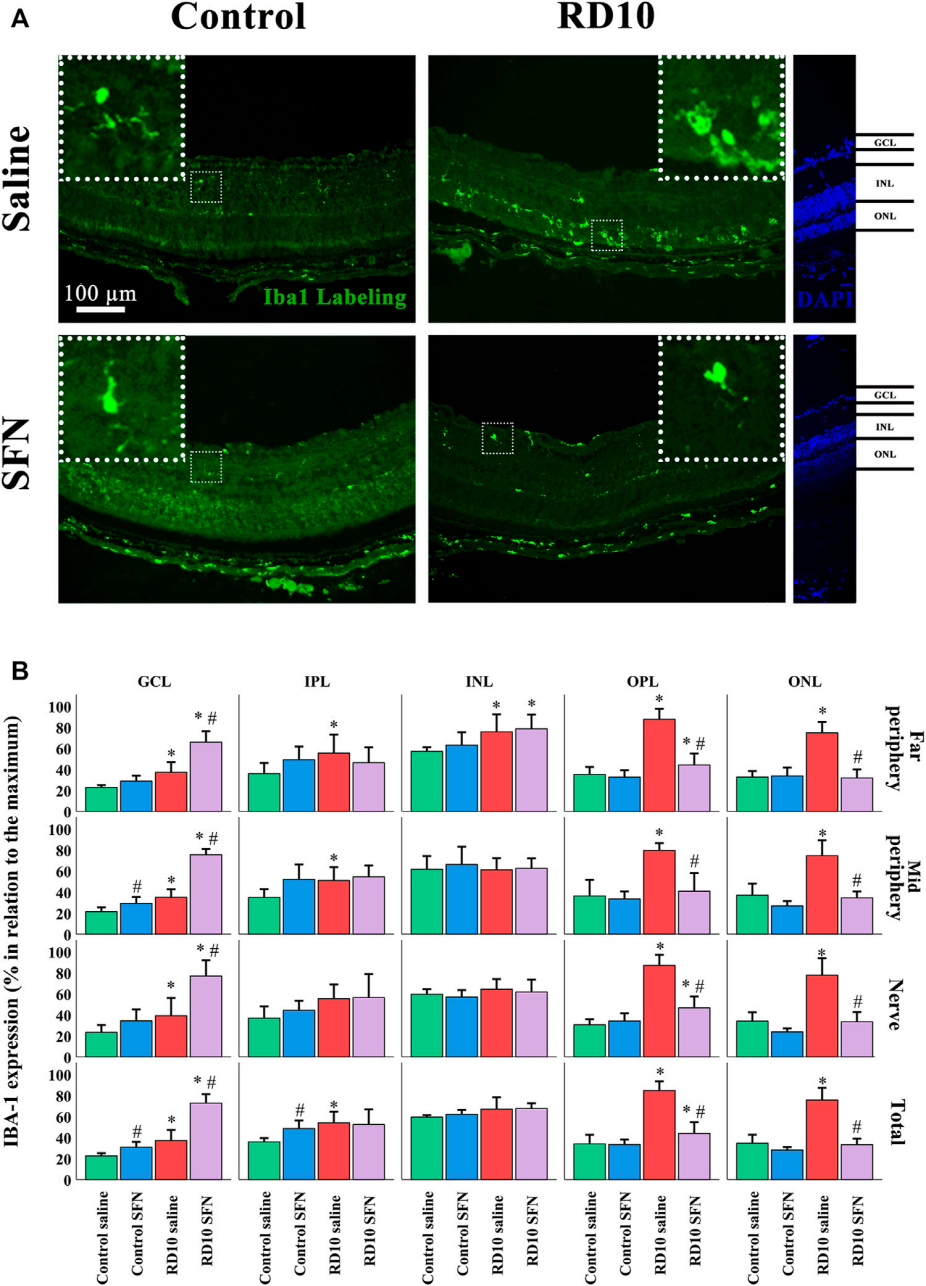
Microglia phenotype analysis **(A)** Nerve region images of Iba1 immunofluorescence of the different groups in the experiment. **(B)** Iba1 cell percentage. *Differences between Control Saline vs. RD10 Saline or Control SFN vs. RD10 SFN (*p*-value <0.05) #Differences between Control Saline vs. Control SFN or RD10 Saline vs. RD10 SFN (*p*-value <0.05).

Microglia expression was analyzed with the classical microglia marker Iba1, which expression was elevated in both RD10 groups in the outer nuclear layer (ONL) (Control Saline vs. RD10 Saline, *p*-value <0.000, Control SFN vs. RD10 SFN, *p* < 0.000). These differences were similar in the outer plexiform layer (OPL) (Control Saline vs. RD10 Saline *p*-value <0.000, Control SFN vs. RD10 SFN, *p* = 0.035). This effect was reverted by the SFN treatment in the RD10 group, in the three regions analyzed (ONL, significant differences between both RD10 groups, *p* < 0.000 in every studied region, OPL, significant differences between both RD10 groups, *p* < 0.000, in every studied region). In the ONL, no differences were detected between the RD10 SFN and control groups, while in the OPL, a slight increase was found concerning the control values (to see all statistical *p*-values, please view the Supplementary Section of the manuscript). Most of the positive Iba1 marks were found in the ONL and OPL layers, but not all. For example, the inner layers, both nuclear (INL) and plexiform (IPL), and the ganglion cell layers (GCL) were positive also to Iba1, and the analysis reveals no differences in the INL between the RD10 groups and the control groups, except in the nerve region. The tendency showed in the previous layers was swift in the GCL, where both RD10 groups showed significant increase with respect to the control groups, but also between the RD10 groups being the RD10 SFN, the one that showed a higher increase (GCL, significant differences between both RD10 groups, *p* < 0.000, in every studied region). The Iba1 results are illustrated in Figure 5.

**FIGURE 6:**
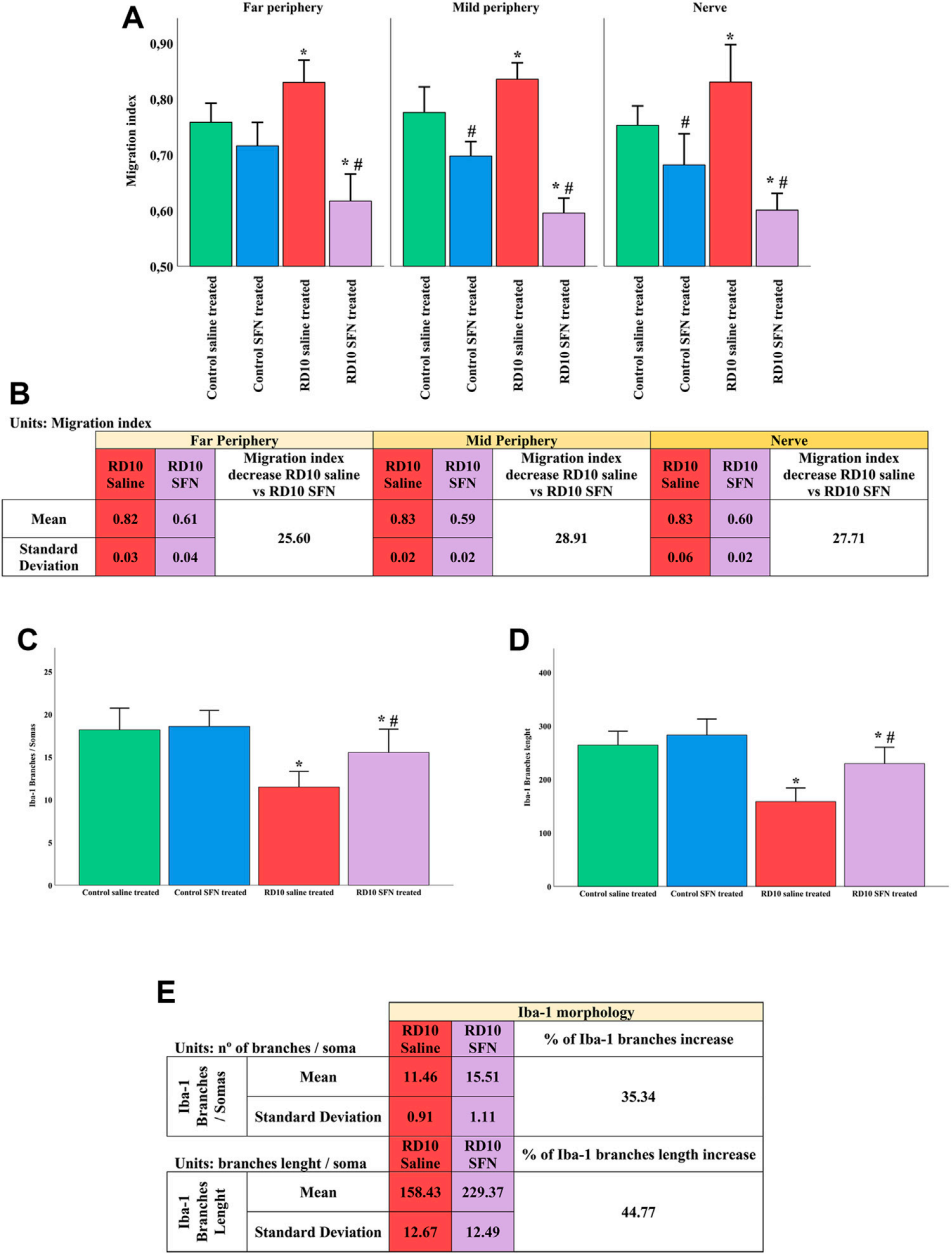
Microglia activation analysis. **(A)** Migration index. **(B)** Migration index: Mean and standard deviation of RD10 Saline and migration index RD10 SFN groups, and percentage decrease in RD10 SFN with respect to the RD10 Saline. **(C)** Branches of Iba1 cells per soma. **(D)** Branch length of Iba1 cells. *Differences between Control Saline vs. RD10 Saline or Control SFN vs. RD10 SFN (*p*-value <0.05). #Differences between RD10 Saline vs. RD10 SFN (*p*-value <0.05). **(E)** Iba1 morphology: Mean and SD of RD10 Saline and RD10 SFN groups, and percentage increase in RD10 SFN vs. RD10 Saline.

Further analysis of the Iba1 marker was carried out to assess the activation and migration of the microglia. Microglia activation is characterized by its migration pattern, from the GCL to the ONL, and morphological changes, such as a length reduction of the branches and soma/projections ratio. The migration index indicated significant increases in the RD10 Saline group in comparison with the control groups (in the far periphery *p* = 0.010, mid periphery *p* = 0.007, nerve region *p* = 0.016), which was reverted by SFN treatment (significant differences between both RD10 groups, *p* < 0.000, far periphery; *p* < 0.000, mid periphery; *p* < 0.000, nerve region). The migration index results are illustrated in Figure 6A, including the mean decrease in RD10 SFN in comparison with the RD10 Saline group (Figure 6B). All the *p*-values can be seen in the Supplementary Data of the manuscript.

The morphological analysis of the microglia indicated a reduction in the microglia branches/soma ratio in the RD10 Saline group (in comparison with the Control Saline group, *p* < 0.000) that was partially reverted by the SFN treatment (significant differences between RD10 groups, *p* = 0.013). These results are presented in Figure 6C, including the mean decrease in RD10 SFN in comparison with the RD10 Saline group (Figure 6E). Finally, complementary to the previous data, the length assessment of the microglia branches indicated a reduction in the RD10 Saline group, which was partially reversed by the SFN treatment (significant differences between RD10 Saline group and Control Saline group, *p* < 0.000, and between RD10 groups, *p* = 0.001). These data are presented in Figure 6D. All statistical *p*-values can be seen in the Supplementary Data of the manuscript.

### Sulforaphane Reduces the Expression of Inflammatory Markers

Microglia and Müller cell activation is characterized by the synthesis and secretion of inflammatory mediators. In this study, we selected and analyzed some of the most relevant inflammatory mediators associated with glial cell activation, including M2 alternative microglia markers. The insight of this analysis was to unveil part of the inflammatory activation pattern of the RD10 Saline group and analyze the SFN effects over this pattern.

**FIGURE 7:**
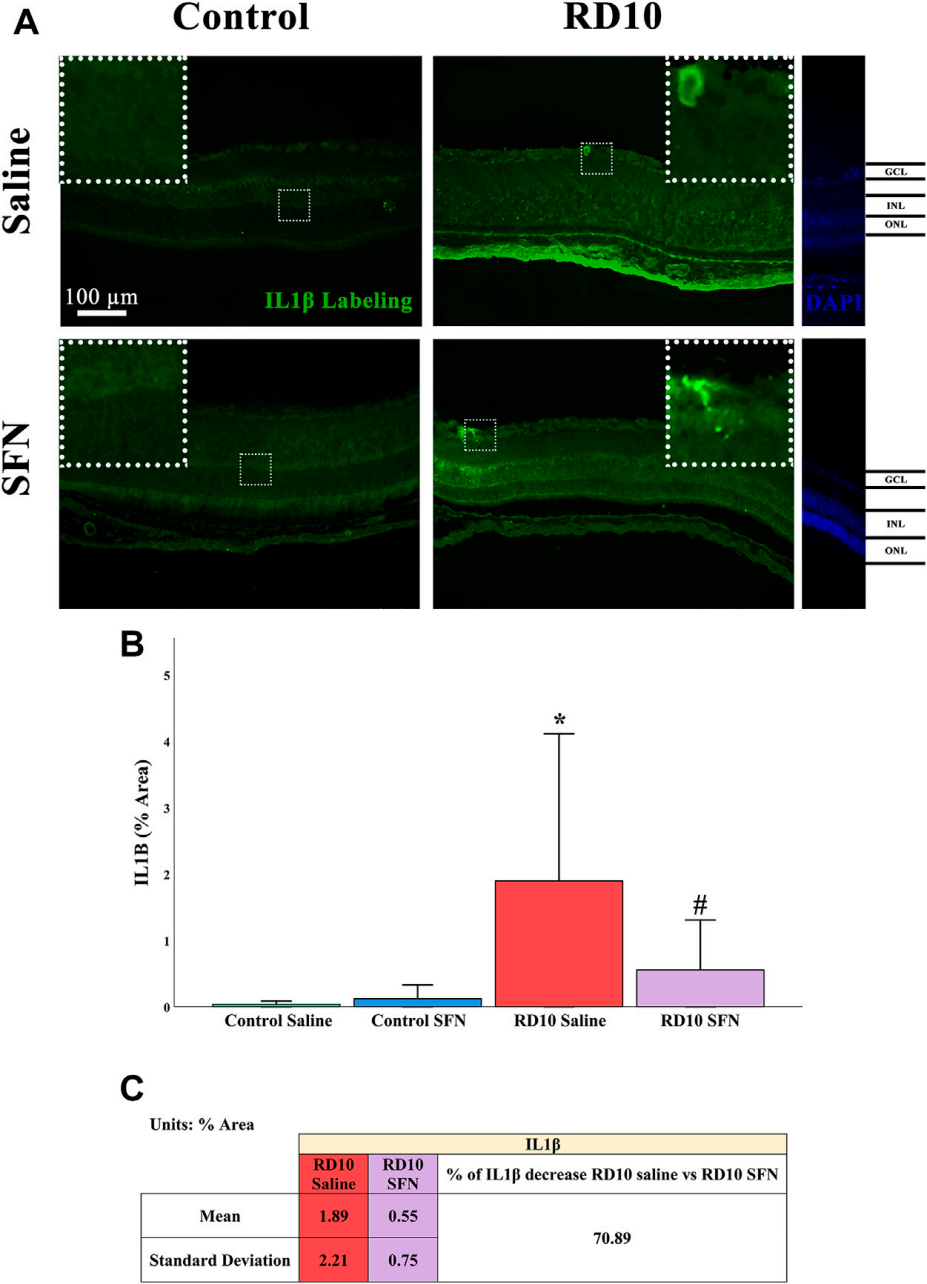
IL1β expression assessment. **(A)** Nerve region images of IL1β immunofluorescence of the different groups in the experiment; white arrows point out the positive mark. **(B)** IL-1β quantification expressed as area percentage. *Differences between Control Saline vs. RD10 Saline (*p*-value <0.05). #Differences between RD10 Saline vs. RD10 SFN (*p*-value <0.05). **(C)** Effect of SFN on RD10 mice IL1β mark expressed by the mean, and standard deviation of the RD10 groups, and percentage increase in RD10 SFN vs. RD10 Saline.

IL1β is considered a proinflammatory mediator (Mendiola and Cardona, 2018; Wooff et al., 2019). Immunofluorescence analysis indicated a significant increase in IL1β-positive area in the RD10 Saline group in comparison with the Control Saline group (*p* = 0.009), which was reversed by the SFN treatment (RD10 Saline vs. RD10 SFN, *p* = 0.051). The IL1β results are illustrated in Figure 7, including the mean decrease in RD10 SFN in comparison with the RD10 Saline group (Figure 7C).

**FIGURE 8:**
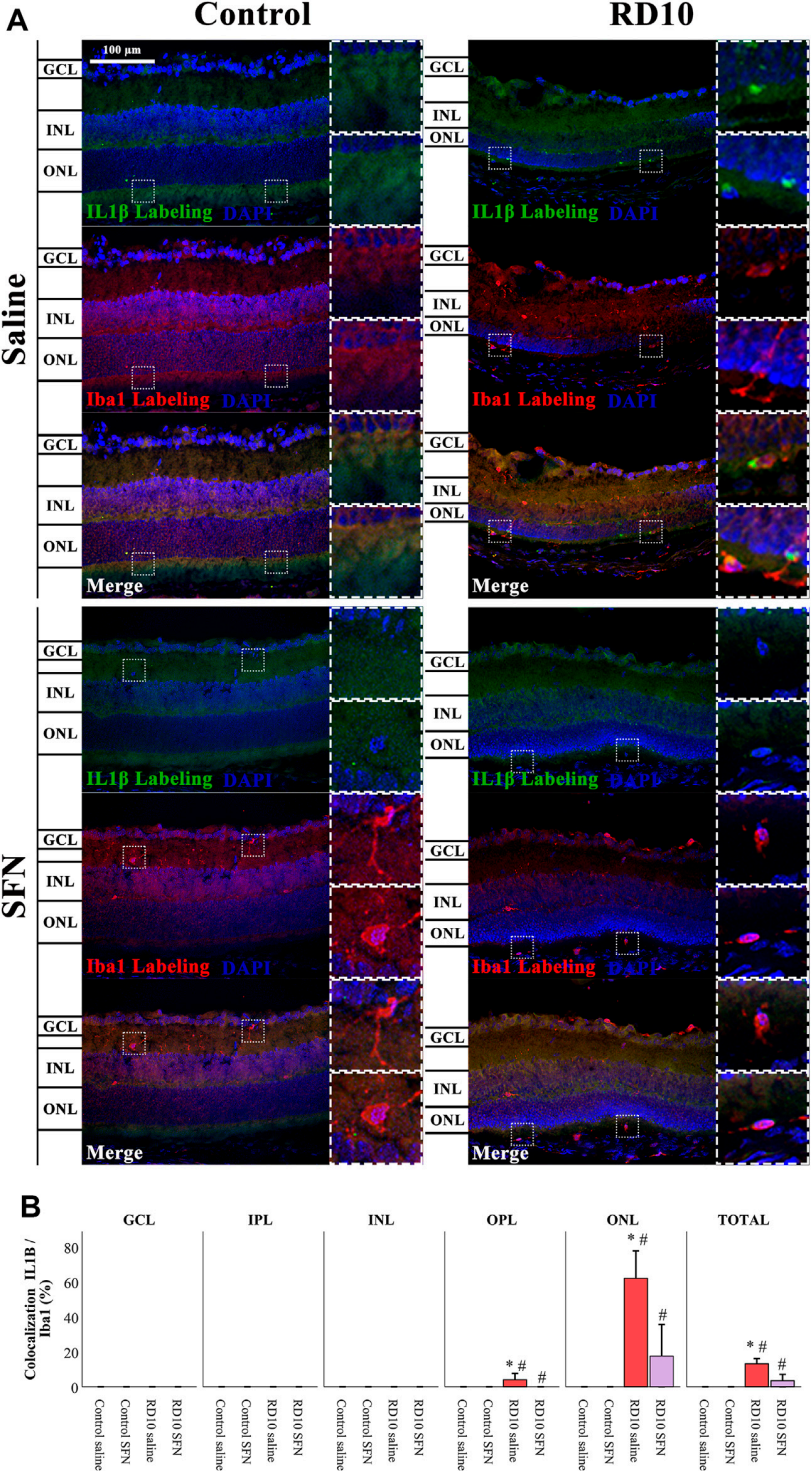
IL1β-Iba1 expression assessment. **(A)** Nerve region images of IL1β-Iba1 immunofluorescence of the different groups in the experiment. **(B)** The percentage of IL1β-Iba1 colocalization was calculated regarding Iba1 total expression. *Differences between Control Saline vs. RD10 Saline or Control SFN vs. RD10 SFN (*p*-value <0.05). #Differences between RD10 Saline vs. RD10 SFN (*p*-value <0.05).

IL1β can be expressed by neurons and glial cells. Colocalization analysis of Iba1 and IL1β was performed to analyze the pattern of microglia IL1β expression based on the recently reported SFN effects as microglia modulator. The analysis reveals that the microglia positive to IL1β was found in the outer layers of the retina. Results indicate a significant increase in IL1β in the ONL of the RD10 Saline group (with respect to the comparison with the Control Saline group, *p* < 0.000), which was reversed by the SFN (differences between RD10 groups, *p* = 0.002). The same profile, but less evident, was displayed in the OPL. The IL1β-Iba1 colocalization results are illustrated in Figure 8. All statistical *p*-values can be seen in the Supplementary Data of the manuscript.

**FIGURE 9:**
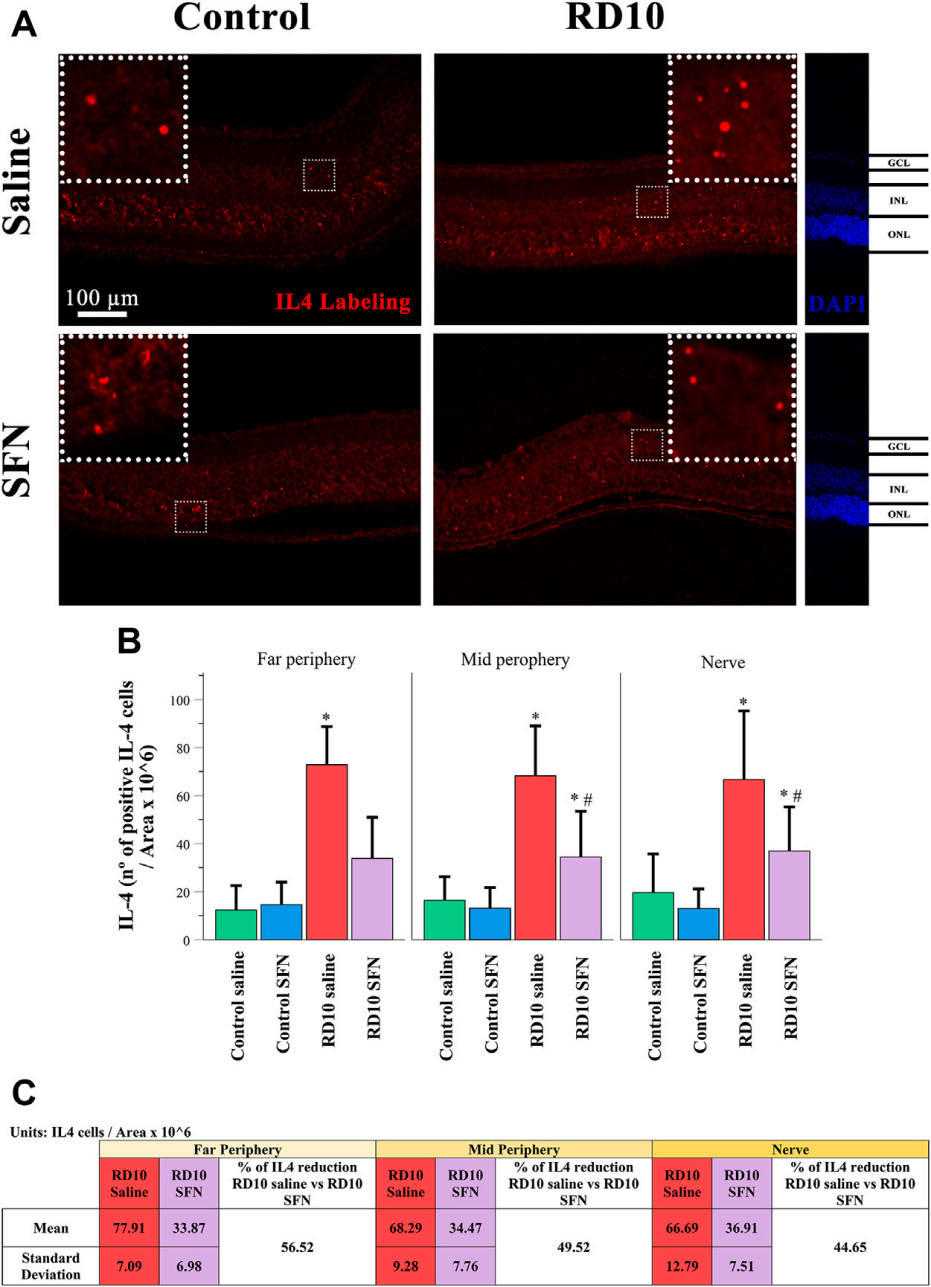
IL-4 expression assessment. **(A)** Nerve region images of IL-4 immunofluorescence of the different groups in the experiment; white arrows point out the positive mark. **(B)** The number of IL-4-positive cells was divided per area and multiplied by 10^6^. *Differences between Control Saline vs. RD10 Saline or Control SFN vs. RD10 SFN (*p*-value <0.05). #Differences between RD10 Saline vs. RD10 SFN (*p*-value <0.05). **(C)** Effect of SFN on RD10 mice IL4 mark expressed by the mean and standard deviation of the RD10 groups, and percentage reduction of RD10 Saline vs. RD10 SFN.

**FIGURE 10:**
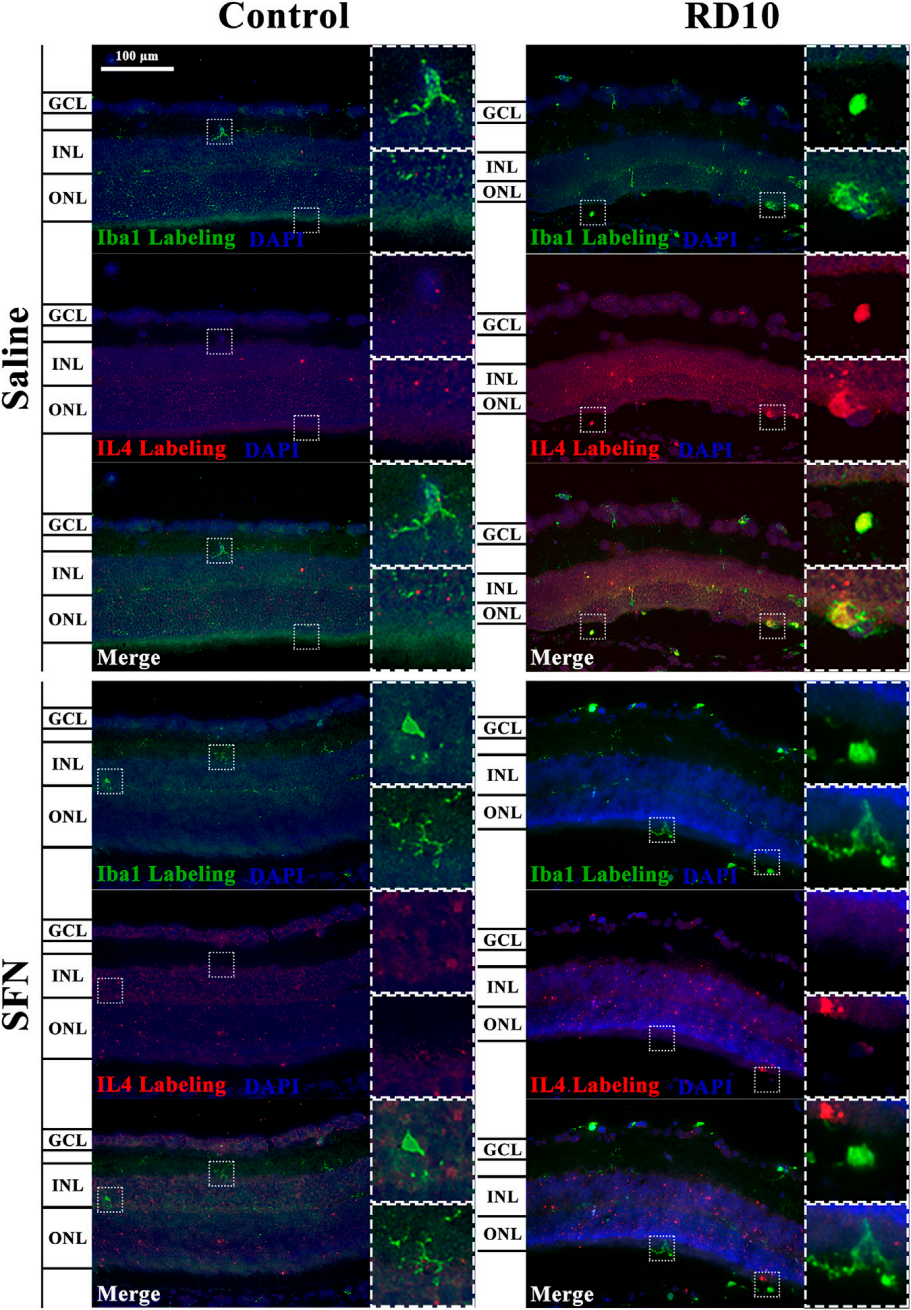
Illustrative IL4-Iba1 immunofluorescent images from the nerve region of the different groups in the experiment.

IL4 has been associated with alternative microglia activation. The immunofluorescence analysis indicated a significant increase in the IL4 expression in the RD10 Saline group (in comparison with the Control Saline group, in the far periphery, *p* = 0.004; mid periphery, *p* = 0.003; and nerve region, *p* = 0.014). This effect was partially reversed by the SFN treatment (comparison between both RD10 groups, *p* = 0.023, far periphery; *p* = 0.007, mid periphery; *p* = 0.038, and nerve region). The IL4 results are illustrated in Figure 9, including the mean decrease in RD10 SFN in comparison with the RD10 Saline group (Figure 9C). All statistical *p*-values can be seen in the Supplementary Data of the manuscript. Further IL4/Iba1 colocalization analysis indicated that IL4-positive cells colocalized with Iba1-positive cells with ameboid shape (Figure 10).

**FIGURE 11:**
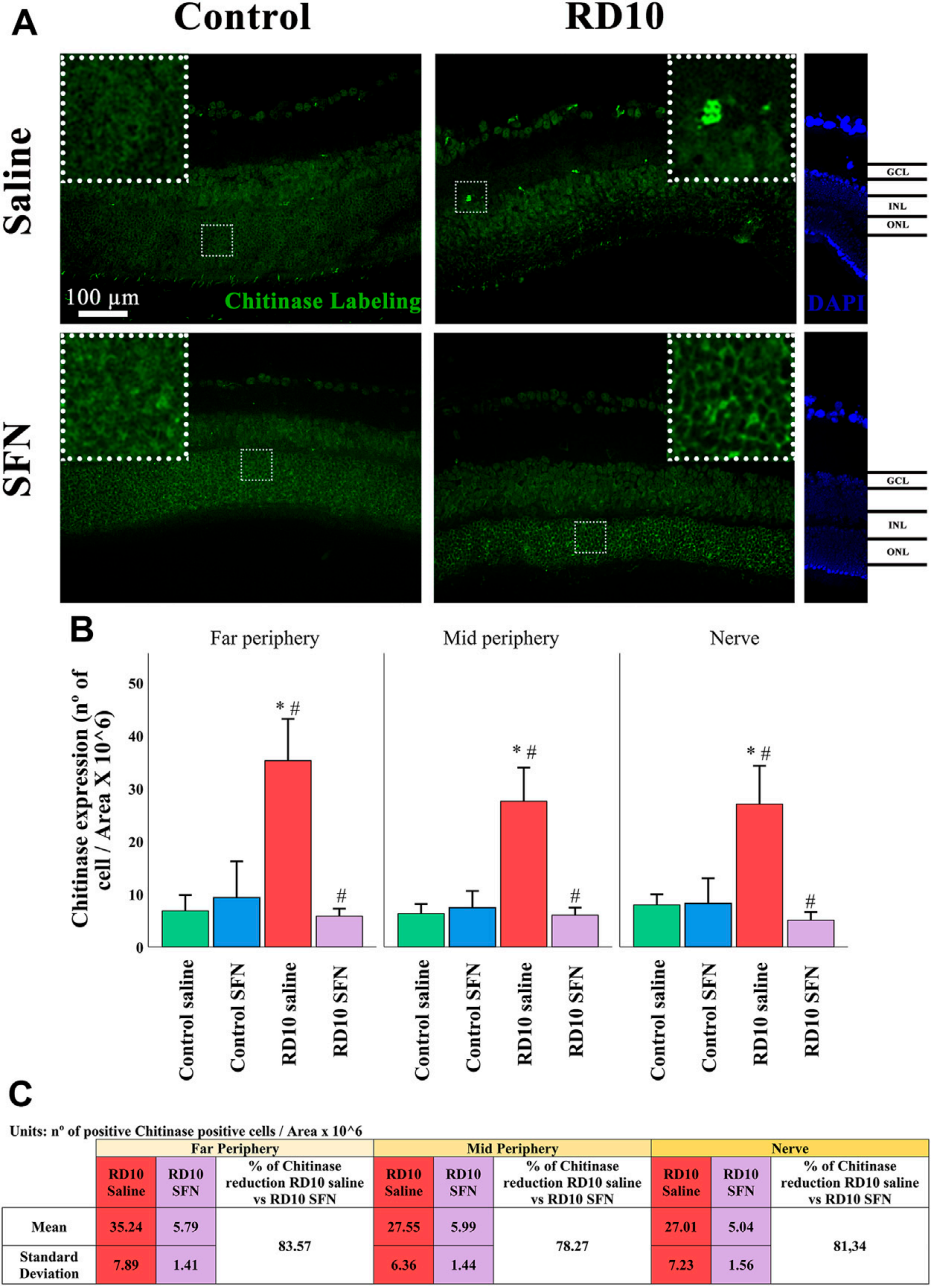
Chitinase 3-like protein (YM1) expression assessment. **(A)** Nerve region images of chitinase immunofluorescence of the different groups in the experiment; white arrows point out the positive mark. **(B)** The number of chitinase-positive cells was divided per area and multiplied by 10^6^. *Differences between Control Saline vs. RD10 Saline or Control SFN vs. RD10 SFN (*p*-value <0.05). #Differences between RD10 Saline vs. RD10 SFN (*p*-value <0.05). **(C)** Effect of SFN on RD10 mice chitinase mark expressed by the mean and standard deviation of the RD10 groups, and percentage reduction of RD10 Saline vs. RD10 SFN.

YM1 (chitinase 3-like protein 3) recognizes a lectin family, which is secreted by macrophages during inflammation. The immunofluorescence analysis showed a significant increase in the RD10 Saline group that was downregulated in the RD10 SFN group (in comparison with the Control Saline group, *p* < 0.001; in comparison with the RD10 SFN, *p* < 0.000). The YM1 results are illustrated in Figure 11, including the mean decrease in RD10 SFN in comparison with the RD10 Saline group (Figure 11C). All statistical *p*-values can be seen in the Supplementary Data of the manuscript.

**FIGURE 12:**
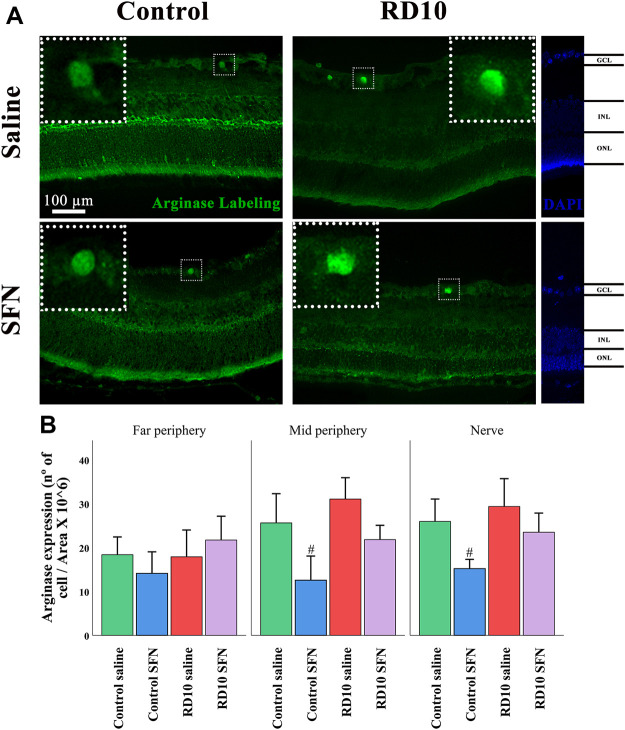
Arginase expression assessment. **(A)** Nerve region images of arginase immunofluorescence of the different groups in the experiment; white arrows point out the positive mark. **(B)** The number of arginase-positive cells was divided per area and multiplied by 10^6^. #Differences between Control Saline vs. Control SFN (*p*-value <0.05).

Finally, the enzyme arginase converts L-arginine to urea and L-ornithine, and its excessive expression has been related to neural toxicity (Caldwell et al., 2015). The immunofluorescence analysis indicated no statistical differences between the RD10 groups and the Control Saline group. However, a significant reduction was found in the Control SFN group in comparison with the Control Saline group (in the mid periphery, *p* = 0.016, and nerve region, *p* = 0.024, but not in the far periphery, *p* = 0.343). The higher mean values were found in the RD10 Saline in the mid periphery (mean = 31.06 cells/area) and nerve region (mean = 29.37 cells/area). The arginase results are illustrated in Figure 12. All statistical *p*-values can be seen in the Supplementary Data of the manuscript.

## Discussion

Our results show that a daily administration of SFN delays the neurodegeneration and reduces the retina inflammation in an animal model of RP. The neuroprotective SFN role on the retina has been documented by other authors, including the retinal function recovery in an animal model of RP (Kang and Yu, 2017). However, how this action is achieved, and the underlying mechanisms, remain uncertain.

The oxidative stress modulation by SFN is the most recognized action mechanism. Concerning RP research, SFN inhibition of endoplasmic reticulum stress has been proposed as a possible mechanism (Kang and Yu, 2017). The antioxidant properties of SFN are well described, as well as the deleterious relevance of oxidative stress over RP development. The death of the first photoreceptor cells would trigger a sequence of oxidative reactions, which accelerate cellular degeneration (Campochiaro and Mir, 2018). SFN neuroprotective effects will interfere with this xenobiotic cascade, by the induction of the transcription of antioxidant enzymes, through the Nrf2/ARE pathways (Gao and Talalay, 2004). However, oxidative stress is a consequence of specific cellular actions, carried out by specific cells, a process intertwined with other cellular actions, all grouped in a most general and comprehensive concept called inflammation (Medzhitov, 2008). In this line, oxidative stress and inflammation are both cellular defense responses to harmful stimuli, which, under persistent and uncontrolled conditions, may amplify the cellular damage. Our results indicated that SFN administration modulates retinal inflammation, and this effect may reduce the associated neurodegeneration.

Neuroinflammation is characterized by glial cell activation. Our data showed a clear Müller cell reaction that was reduced by SFN administration (see Figure 4). Similar results have been described in diabetic retinopathy (Li et al., 2019), where the SFN protection over the Müller cells was associated with the activation of the Nrf2 pathway and the inhibition of the inflammasome. Moreover, the interplay between Müller cells and microglia has been described in eye pathologies, by which the activation of both glial cells induces continuous activation feedback (Hu et al., 2021). A direct SFN effect over the Müller cells or an indirect effect through the microglia SFN modulation should be considered as underlying mechanisms.

Microglia are macrophages resident in the nervous system, which plays a main role during the inflammation reaction. The activation of the microglia is characterized by the migration, proliferation, morphology changes, and expression and secretion of inflammatory mediators. Our cellular microglia analysis indicates a microglia activation in the RD10 Saline group. This statement is supported by the increased migration index, the increased expression of Iba1, mainly in the ONL, as well as the length reduction of the projections and the soma/projections ratio in the Iba1 cells (see Figures 5, 6). These effects were reduced by the SFN treatment in the RD10 SFN group, but no effect was detected in the Control SFN group. SFN anti-inflammatory properties have been shown in several publications. Proposed mechanisms include the activation of anti-inflammatory genes, the modulation of internal cellular pathways, such as MAP-Kinase p38 (Subedi et al., 2019), or the inhibition of inflammasome (Tufekci et al., 2021). The data suggest that the SFN administration on the Control group does not induce an inflammatory response. The significant H&E reduction induced by the SFN in the Control SFN group is striking in comparison with the Control Saline group. However, the reduction is minimal; in any case, further analysis should explore these data on healthy individuals. Moreover, no differences between Control groups were detected by TUNEL analysis, indicating no SFN noxious effects on cell survival (see Figures 2, 3).

The term inflammation groups cellular and molecular reactions, including the overexpression of several mediators. To complement the neuroinflammation analysis, the expression of a selected group of inflammatory mediators was analyzed. In the animal model of RP, at 21 days of age, the results indicate an increased expression of IL1β, IL4, and YM-1 that was reduced by SFN.

IL1β has been described as a relevant proinflammatory marker, involved in several processes, such as neutrophil recruitment, inflammatory mediator, inflammasome activator, or even in angiogenesis. The reported IL1β elevation in the rd10 model indicates an inflammatory process, which is reduced by the SFN treatment. The Iba1 colocalization suggests a link between the expression of the cytokine and the activation of the microglia that is not expressed in the group with SFN (see Figures 7, 8). These results indicate that the SFN treatment reduced the inflammatory IL1β pattern shown in the rd10 model, and based on the colocalization data, one of the consequences seems to be the modulation of the microglia activation. It is a highlight that the colocalization analysis indicates a strong colocalization in the ONL, where the Iba1 expression was detected higher. It is relevant to add that the IL1β marker used in this study (Abcam, ab9722) reacts with two IL1β isoforms, the mature and the pro-form. For this reason, it cannot be discriminated which of both isoforms may be affected by SFN This issue should be considered in future studies.

IL4 is a cytokine that displays several functions, which are different based on the glia cell type analyzed and the IL4 concentration (Brodie and Goldreich, 1994). IL4 expression has been associated with M2 microglia polarization, as well as with eye pathological conditions (Zandi et al., 2019; Chen et al., 2021). The results indicate an increased expression of IL4 in the RD10 Saline group, which was reduced by the SFN treatment. IL4 was expressed in microglia cells, although its expression in other cell types cannot be ruled out (see Figures 9 **and** 10). By its side, YM1 is secreted by alternatively activated macrophages. Its elevation has been related to some eye pathologies (Rojas et al., 2014). The YM1 expression analysis shed similar results to previous cytokines. It was elevated in the RP animal model and reduced by the SFN treatment (See Figure 11). Finally, arginase is an enzyme that hydrolyzes the amino acid, L-arginine, to ornithine and urea, and is activated during neuroinflammation. It has been reported that a disproportionate arginase activation is associated with neurotoxicity (Caldwell et al., 2015). Our results do not indicate significant differences in arginase expression, except a significant reduction in the Control SFN group. It should be noted that the greater mean value was registered in the RD10 Saline group (See Figure 12). Together, these results suggest that RP runs parallel with an inflammation process, which implies glial cell activation, and that the SFN treatment may modulate this inflammation response, a fact connected with its neuroprotective properties. Moreover, based on our data, this modulation seems relevant in microglial cells, without discarding Müller cell regulation.

We are fully aware that IL4, YM1, and arginase are markers of the microglia M2 polarization state. The study of M1/M2 microglia phenotype is not within the scope of this article. However, it is relevant to consider the surprising increased expression of these inflammatory markers. Besides the microglia M2 phenotype, the expression and activity of these molecules have also been related to neuroinflammation and neurotoxicity, suggesting alternative actions to the M2 repair phenotype. Moreover, the M1/M2 phenotypes are conditioned by the disease progression state (Arroba et al., 2016). The SFN treatment modulates the microglia activation and possibly the polarization timing. Without a doubt, future analyses of the microglia polarization in RP will shed light on this issue, but our results indicate a microglia activation, including the upregulation of classical M2 markers in rd10 mice, and its reversion by SFN.

Reaching to this point, the data indicated an inflammatory SFN regulation. However, how does the SFN regulate the microglia and Müller cell activation state? This is a precise and relevant question.

It is logical to propose that the SFN effect over the glia cells would be carried out through the well-described antioxidant Nrf2/ARE pathway, inducing the transcription of genes related to the anti-inflammatory glial cell phenotypes. However, additional and independent Nrf2/ARE SFN intracellular pathways have been described, including inhibition of the inflammasome (Greaney et al., 2016), activation of lysosome and autophagosome programs (Li et al., 2021), and regulation of intracellular architecture (Zhou et al., 2018), which must be considered. Further research is required to unveil the SFN mechanisms alternative to the antioxidant Nrf2/ARE pathway.

Besides acting by direct intracellular pathways, the SFN anti-inflammatory properties, such as microglia phenotype regulation, may be carried out through the astrocyte–microglia homeostasis modulation (Kim and Son, 2021). Our results indicate that the SFN reduces both microglia cell and Müller cell activations. However, are both effects triggered directly by SFN or by glia interactions? Nrf2 is expressed at high levels in astrocytes, pointing out Müller cells as a relevant cellular target of SFN treatment (Navneet et al., 2019; Xu et al., 2019). Moreover, interleukin 1β has been proposed as an intercellular mediator in the regulation of both glial cells (Natoli et al., 2017), and its expression and secretion can be modulated by SFN (Hernandez-Rabaza et al., 2016). Glial cell interactions are required to be understood to further comprehend the SFN mechanism.

To summarize, the main idea that arises from our results is that the SFN reduces retinal neurodegeneration, and this effect would be accomplished, in part, by the anti-inflammatory SFN properties. The regulation of neuroinflammation contributes to achieving neuroprotection in diseases where the harmful stimuli are persistent. Our data supported this statement in the case of SFN and RP. In addition, the microglia activation pattern modulation by SFN treatment emerges as a relevant therapeutic target. In this line, further research is required to understand the underlying SFN mechanism over intracellular and intercellular glia regulations.

## Data Availability

The raw data supporting the conclusion of this article will be made available by the authors, without undue reservation.
