# Supplementary material for: Sulforaphane Modulates the Inflammation and Delays Neurodegeneration on a Retinitis Pigmentosa Mice Model
Source: Front Pharmacol. 2022 Mar 1;13:811257. doi: 10.3389/fphar.2022.811257 (PMC8921528; doi:10.3389/fphar.2022.811257)
Supplement: Supplementary file 1 [file DataSheet1.PDF]

Supplementary statistical information: p-values

The two-way analysis of variance (ANOVA) was used. When the ANOVA indicated a significant difference, the Bonferroni test was performed. SPSS software package version 27.0 was used. In every case, it was assumed a p-value lower than 0.05.

Eosine-hematoxilin		
**ZONE**	**GROUPS COMPARED**	**p-value**
Far periphery	Control Saline-Control SFN	=0,175
	RD10 Saline-RD10 SFN	=0,062
	Control Saline-RD10 Saline	<0,000
	Control SFN- RD10 SFN	=0,001
Mid periphery	Control Saline-Control SFN	=0,010
	RD10 Saline-RD10 SFN	<0,000
	Control Saline-RD10 Saline	<0,000
	Control SFN- RD10 SFN	<0,000
Nerve	Control Saline-Control SFN	=0,037
	RD10 Saline-RD10 SFN	<0,000
	Control Saline-RD10 Saline	<0,000
	Control SFN- RD10 SFN	<0,000

TUNEL		
**ZONE**	**GROUPS COMPARED**	**p-value**
Far periphery	Control Saline-Control SFN	=0,839
	RD10 Saline-RD10 SFN	<0,000
	Control Saline-RD10 Saline	<0,000
	Control SFN- RD10 SFN	<0,000
Mid periphery	Control Saline-Control SFN	=0,813
	RD10 Saline-RD10 SFN	<0,000
	Control Saline-RD10 Saline	<0,000
	Control SFN- RD10 SFN	<0,000
Nerve	Control Saline-Control SFN	=0,955
	RD10 Saline-RD10 SFN	<0,000
	Control Saline-RD10 Saline	<0,000
	Control SFN- RD10 SFN	<0,000

GFAP		
**ZONE**	**GROUPS COMPARED**	**p-value**
Far periphery	Control Saline-Control SFN	=0,812
	RD10 Saline-RD10 SFN	<0,000
	Control Saline-RD10 Saline	<0,000
	Control SFN- RD10 SFN	<0,000
Mid periphery	Control Saline-Control SFN	=0,695
	RD10 Saline-RD10 SFN	<0,000
	Control Saline-RD10 Saline	<0,000
	Control SFN- RD10 SFN	<0,000

Nerve	Control Saline-Control SFN	=0,344
	RD10 Saline-RD10 SFN	<0,000
	Control Saline-RD10 Saline	<0,000
	Control SFN- RD10 SFN	<0,000

IBA1		
**LAYER IN "TOTAL" ZONE**	**GROUPS COMPARED**	**p-value**
GCL	Control Saline-Control SFN	=0,044
	RD10 Saline-RD10 SFN	<0,000
	Control Saline-RD10 Saline	=0,002
	Control SFN- RD10 SFN	<0,000
IPL	Control Saline-Control SFN	=0,031
	RD10 Saline-RD10 SFN	=0,799
	Control Saline-RD10 Saline	=0,006
	Control SFN- RD10 SFN	=0,486
INL	Control Saline-Control SFN	=0,453
	RD10 Saline-RD10 SFN	=0,876
	Control Saline-RD10 Saline	=0,053
	Control SFN- RD10 SFN	=0,115
OPL	Control Saline-Control SFN	=0,896
	RD10 Saline-RD10 SFN	<0,000
	Control Saline-RD10 Saline	<0,000
	Control SFN- RD10 SFN	=0,035
ONL	Control Saline-Control SFN	=0,133
	RD10 Saline-RD10 SFN	<0,000
	Control Saline-RD10 Saline	<0,000
	Control SFN- RD10 SFN	=0,227

Migration index		
**ZONE**	**GROUPS COMPARED**	**p-value**
Far periphery	Control Saline-Control SFN	=0,083
	RD10 Saline-RD10 SFN	<0,000
	Control Saline-RD10 Saline	=0,010
	Control SFN- RD10 SFN	<0,000
Mid periphery	Control Saline-Control SFN	<0,000
	RD10 Saline-RD10 SFN	<0,000
	Control Saline-RD10 Saline	=0,007
	Control SFN- RD10 SFN	<0,000
Nerve	Control Saline-Control SFN	=0,016
	RD10 Saline-RD10 SFN	<0,000
	Control Saline-RD10 Saline	=0,016
	Control SFN- RD10 SFN	=0,007

Microglia branches/soma ratio	
**GROUPS COMPARED**	**p-value**
Control Saline-Control SFN	=0,750
RD10 Saline-RD10 SFN	=0,013
Control Saline-RD10 Saline	<0,000
Control SFN- RD10 SFN	=0,028

Microglia branches lenght	
**GROUPS COMPARED**	**p-value**
Control Saline-Control SFN	=0,225
RD10 Saline-RD10 SFN	=0,001
Control Saline-RD10 Saline	<0,000
Control SFN- RD10 SFN	=0,003

IL1β	
**GROUPS COMPARED**	**p-value**
Control Saline-Control SFN	=0,890
RD10 Saline-RD10 SFN	=0,051
Control Saline-RD10 Saline	=0,009
Control SFN- RD10 SFN	=0,474

IL1β - IBA1 ONL colocalization	
**GROUPS COMPARED**	**p-value**
Control Saline-Control SFN	=1,000
RD10 Saline-RD10 SFN	=0,002
Control Saline-RD10 Saline	<0,000
Control SFN- RD10 SFN	=0,110

IL4		
**ZONE**	**GROUPS COMPARED**	**p-value**
Far periphery	Control Saline-Control SFN	=0,266
	RD10 Saline-RD10 SFN	=0,023
	Control Saline-RD10 Saline	=0,004
	Control SFN- RD10 SFN	=0,055
Mid periphery	Control Saline-Control SFN	=0,403
	RD10 Saline-RD10 SFN	=0,007
	Control Saline-RD10 Saline	=0,003
	Control SFN- RD10 SFN	=0,193
Nerve	Control Saline-Control SFN	=0,119
	RD10 Saline-RD10 SFN	=0,038
	Control Saline-RD10 Saline	=0,014
	Control SFN- RD10 SFN	=0,044

YM1		
**ZONE**	**GROUPS COMPARED**	**p-value**
Far periphery	Control Saline-Control SFN	=0,591
	RD10 Saline-RD10 SFN	<0,000
	Control Saline-RD10 Saline	<0,000
	Control SFN- RD10 SFN	=0,452
Mid periphery	Control Saline-Control SFN	=0,719
	RD10 Saline-RD10 SFN	<0,000
	Control Saline-RD10 Saline	<0,000
	Control SFN- RD10 SFN	=0,655
Nerve	Control Saline-Control SFN	=0,942
	RD10 Saline-RD10 SFN	<0,000
	Control Saline-RD10 Saline	<0,001
	Control SFN- RD10 SFN	=0,415

Arginase		
**ZONE**	**GROUPS COMPARED**	**p-value**
Far periphery	Control Saline-Control SFN	=0,343
	RD10 Saline-RD10 SFN	=0,393
	Control Saline-RD10 Saline	=0,914
	Control SFN- RD10 SFN	=0,110
Mid periphery	Control Saline-Control SFN	=0,016
	RD10 Saline-RD10 SFN	=0,062
	Control Saline-RD10 Saline	=0,238
	Control SFN- RD10 SFN	=0,062
Nerve	Control Saline-Control SFN	=0,024
	RD10 Saline-RD10 SFN	=0,168
	Control Saline-RD10 Saline	=0,405
	Control SFN- RD10 SFN	=0,064
